# Prospective Assessment of Short-Term Response and Acute Toxicity With Accelerated Fractionation Radiotherapy Versus Concurrent Chemoradiotherapy in Locally Advanced Cervical Cancer

**DOI:** 10.7759/cureus.114053

**Published:** 2026-08-06

**Authors:** Pranabandhu Das, GV Deepthi, Praneetha Chevireddy, Bala Venkat Subramanian, Sreenivasa Rao Bonala

**Affiliations:** 1 Radiation Oncology, Sri Venkateswara Institute of Medical Sciences (SVIMS), Tirupati, IND

**Keywords:** accelerated fractionation radiotherapy, cervical cancer, chemoradiotherapy, local response, overall treatment time, treatment toxicity

## Abstract

Objectives: This study aims to compare treatment response, toxicity, and feasibility of accelerated fractionation radiotherapy (AFRT) versus concurrent chemoradiotherapy (CCRT) in locally advanced cervical cancer.

Materials and methods: In this prospective, non-randomized study, 92 patients with International Federation of Gynecology and Obstetrics (FIGO) 2009 stage IB2-IIIB cervical cancer (May 2018-July 2019) were allocated to AFRT (n=44; external beam radiotherapy (EBRT) 50 Gy/25 fractions, six fractions/week, no chemotherapy) or CCRT (n=48; EBRT 50 Gy/25 fractions, five fractions/week, weekly cisplatin 40 mg/m²) based on fitness for concurrent chemotherapy. All received high-dose-rate intracavitary brachytherapy (7 Gy × 3). The primary endpoint was local response; the secondary endpoints were acute and late toxicities. Given the non-randomized allocation, age-adjusted (Firth's penalized where indicated) logistic regression was performed for key endpoints.

Results: The AFRT arm was older (median age: 62.5 versus 51 years), reflecting the selection criteria. The overall treatment time (OTT) was significantly shorter with AFRT (51 versus 56 days; p=0.0043). Complete response at three months was comparable (93.2% versus 95.8%; p=0.57; age-adjusted odds ratio (OR): 1.98, 95% confidence interval (CI): 0.28-14.1, p=0.50). CCRT showed a significantly higher incidence of grade 2 leucopenia (4.5% versus 23%; p=0.01; age-adjusted OR: 9.95, 95% CI: 1.97-50.3, p=0.005), a numerically higher rate of grade 3 leucopenia (0% versus 8%), and higher grade 2 skin toxicity (4.5% versus 18.75%; p=0.03). Acute kidney injury (AKI) occurred exclusively with CCRT (22.9%; p=0.0007; age-adjusted OR: 28.5, 95% CI: 1.60-507, p=0.023), including grade 1 AKI in 14.6% (p=0.008). Gastrointestinal toxicities were comparable (p>0.05). Late toxicities, assessed in a subset of patients (24 AFRT and 22 CCRT) due to short follow-up, showed no significant differences, although wide, overlapping 95% confidence intervals indicate limited power to detect true differences.

Conclusion: In this short-term analysis (median follow-up: 5 months), AFRT achieved response rates comparable to CCRT while significantly shortening treatment time and reducing hematological and renal toxicity, and these associations persisted after age adjustment. Given the non-randomized design, baseline age difference, and short follow-up, findings are hypothesis-generating and pertain to short-term response and acute toxicity rather than long-term outcomes. AFRT may be a feasible option for patients unsuitable for concurrent chemotherapy, meriting further evaluation in larger randomized studies with longer follow-up.

## Introduction

Cervical cancer remains a major global health problem, ranking as the fourth most common cancer among women worldwide, with 662,044 new cases and 348,709 deaths reported in 2022 according to GLOBOCAN [1]. In India, it continues to be a significant public health burden, accounting for 127,526 new cases and 79,906 deaths annually [2].

Radiotherapy with external beam radiotherapy (EBRT) and intracavitary brachytherapy is the cornerstone of treatment for locally advanced cervical cancer. Since the National Cancer Institute alert in 1999 [3], multiple randomized trials and meta-analyses have demonstrated improved survival with cisplatin-based concurrent chemoradiotherapy (CCRT), establishing it as the standard of care [4,5]. However, chemotherapy-related toxicities such as nephrotoxicity and myelosuppression limit its applicability in elderly patients and those with renal dysfunction, comorbidities, or poor performance status.

For patients unsuitable for chemotherapy, alternative treatment strategies are required. Altered fractionation radiotherapy has been investigated to improve outcomes in patients unsuitable for chemotherapy; however, hyperfractionation has shown limited clinical benefit with increased toxicity [6]. In contrast, accelerated fractionation shortens overall treatment time (OTT), thereby reducing tumor repopulation, an important cause of treatment failure in cervical cancer [7,8].

Accelerated fractionation radiotherapy (AFRT), delivered as six fractions per week without changing the total radiation dose, may therefore improve tumor control while avoiding chemotherapy-related toxicity. In view of the limited prospective evidence comparing AFRT with standard CCRT, the present study was undertaken to evaluate the efficacy, toxicity, and feasibility of AFRT as an alternative to CCRT in patients with locally advanced cervical carcinoma.

## Materials and methods

Study design and patients

This prospective, non-randomized study enrolled 117 patients with histopathologically confirmed carcinoma of the cervix (International Federation of Gynecology and Obstetrics (FIGO) 2009 stage IB2-IVA, Eastern Cooperative Oncology Group (ECOG) performance status 0-2) between May 2018 and July 2019, based on predefined inclusion and exclusion criteria. Patients with prior pelvic irradiation, prior hysterectomy, metastatic disease, or poor performance status (ECOG 3-4) were excluded. Fifty-seven patients were initially enrolled in the AFRT arm (Arm A) and 60 in the CCRT arm (Arm B). Thirteen patients from the AFRT arm and 12 from the CCRT arm were subsequently excluded due to ineligibility for intracavitary brachytherapy, protocol non-compliance, or unwillingness to undergo brachytherapy, leaving 44 patients in the AFRT arm and 48 in the CCRT arm for final analysis. Institutional ethics committee approval was obtained from Sri Venkateswara Institute of Medical Sciences, Tirupati (IEC No.: 734; Letter Roc. No.: AS/11/IEC/2017), and written informed consent was secured from all participants.

Pretreatment evaluation

Clinical staging was performed according to FIGO 2009 criteria using detailed gynecological examination. Baseline evaluation included contrast-enhanced CT or MRI of the abdomen and pelvis depending on availability and clinical indication. Baseline evaluation also included chest radiography and laboratory investigations comprising complete blood counts, renal and liver function tests, and viral markers.

Treatment protocol

Patients considered unsuitable for concurrent chemotherapy because of advanced age, impaired renal function (estimated glomerular filtration rate (eGFR) < 60 mL/min), significant comorbidities, poor nutritional status, or physician-assessed inability to tolerate cisplatin were assigned to the AFRT arm, while medically fit patients received standard CCRT.

The AFRT arm (Arm A) received external beam radiotherapy (EBRT) 50 Gy in 25 fractions delivered as six fractions per week without chemotherapy in patients unsuitable for concurrent chemotherapy. The CCRT arm (Arm B) received EBRT 50 Gy in 25 fractions delivered as five fractions per week with weekly cisplatin (40 mg/m²).

All patients received high-dose-rate intracavitary brachytherapy (7 Gy × 3 weekly fractions).

Radiotherapy technique

All patients were treated with three-dimensional conformal radiotherapy (3DCRT) following CT-based planning. Target volumes and organs at risk were contoured as per Radiation Therapy Oncology Group (RTOG) guidelines [9-11]. Treatment plans were optimized to ensure that at least 95% of the planning target volume received 95% of the prescribed dose. All treatment plans underwent institutional quality assurance review before treatment initiation.

Assessment and follow-up

Tumor response was assessed clinically at one and two months after treatment by two independent radiation oncologists. CT/MRI was performed at the second follow-up whenever clinically indicated or available, and radiological response was evaluated using RECIST version 1.1 [12]. Patients without imaging were assessed by standardized clinical examination. Acute toxicities were evaluated weekly using CTCAE version 4.0 [13], and late toxicities, defined as adverse events occurring ≥90 days after completion of radiotherapy, were assessed using the RTOG/EORTC Late Radiation Morbidity Scoring Schema during follow-up visits [14]. Patients were followed up monthly for the first three months, every three months up to one year, and every four months during the second year.

Statistical analysis

Categorical variables were compared using the chi-square test or Fisher's exact test, as appropriate, and continuous variables using Student's t-test. A two-sided p-value <0.05 was considered statistically significant. Because treatment allocation was non-randomized and the treatment arms differed substantially in age, age-adjusted multivariable logistic regression was performed for the primary response endpoint and key acute toxicity outcomes. Firth's penalized logistic regression was used for outcomes with zero events in one treatment arm.

## Results

After all exclusions, 44 patients in the AFRT arm and 48 in the CCRT arm were finally evaluated for response and acute toxicity. The median follow-up duration was five months (range: 2-13 months).

Patient and disease characteristics

The median age was higher in the AFRT arm (62.5 years) compared to the CCRT arm (51 years), with a greater proportion of elderly patients (>60 years) in the AFRT group (59% versus 21%). Stage distribution was comparable between groups, with the majority of patients presenting with FIGO stage II and III disease.

Treatment delivery and overall treatment time

The median EBRT duration was significantly shorter in the AFRT arm compared to the CCRT arm (30 versus 35 days; p<0.00001). Similarly, the median overall treatment time (OTT) was significantly reduced in the AFRT arm (51 versus 56 days; p=0.0043) (Table 1).

**Table 1:** Treatment delivery and overall treatment time EBRT: external beam radiation therapy, OTT: overall treatment time, AFRT: accelerated fractionation radiotherapy, CCRT: concurrent chemoradiotherapy

Parameter	AFRT (n=44)	CCRT (n=48)	p-value
Median EBRT duration (days)	30	35	<0.00001
Median OTT (days)	51	56	0.0043

Local response

At three months post-treatment, complete response rates were comparable between the two groups (AFRT versus CCRT: 93.2% versus 95.8%; p=0.57). Partial response was observed in 6.8% of patients in the AFRT arm and 4.2% in the CCRT arm. No locoregional recurrences were observed during the follow-up period (Table 2).

**Table 2:** Treatment response at three months AFRT: accelerated fractionation radiotherapy, CCRT: concurrent chemoradiotherapy

Response	AFRT (n=44)	CCRT (n=48)	p-value
Complete response	41 (93.2%)	46 (95.8%)	0.57
Partial response	3 (6.8%)	2 (4.2%)	0.66

Age-adjusted analysis

Because treatment allocation was based on clinical fitness rather than randomization and the treatment arms differed substantially in age, age-adjusted multivariable logistic regression was performed for the primary response endpoint and key acute toxicity outcomes. Firth's penalized logistic regression was used for acute kidney injury (AKI) and grade 3 leukopenia because no events occurred in the AFRT arm. In each model, age was not an independent predictor of outcome, and age adjustment yielded treatment effect estimates similar to those observed in the unadjusted analyses (Table 3).

**Table 3:** Age-adjusted multivariable logistic regression analysis of complete response and key acute toxicity outcomes AFRT: accelerated fractionation radiotherapy, CCRT: concurrent chemoradiotherapy, OR: odds ratio, CI: confidence interval

Outcome	Adjusted OR (CCRT versus AFRT)	95% CI	p-value
Complete response	1.98	0.28-14.1	0.50
Grade ≥2 leucopenia	9.95	1.97-50.3	0.005
Acute kidney injury (any grade)	28.5	1.60-507	0.023
Grade 2 skin toxicity	4.87	0.91-26.2	0.065
Grade 3 leucopenia	11.9	0.60-236	0.10

Acute toxicities

Hematological Toxicity

Leucopenia was significantly more frequent in the CCRT arm. Grade 2 leucopenia was observed in 4.5% of AFRT patients compared to 23% in the CCRT group (p=0.01). Grade 3 leucopenia occurred exclusively in the CCRT arm (8%). Hemoglobin toxicity did not differ significantly between groups (Table 4).

**Table 4:** Hematological toxicities (acute) AFRT: accelerated fractionation radiotherapy, CCRT: concurrent chemoradiotherapy

Toxicity	AFRT (n=44)	CCRT (n=48)	p-value
Grade ≥2 anemia	11 (25%)	12 (25%)	1.0
Grade 2 leucopenia	2 (4.5%)	11 (23%)	0.01
Grade 3 leucopenia	0 (0%)	4 (8%)	0.05

Non-hematological Acute Toxicities

Gastrointestinal toxicity: Gastrointestinal toxicities, including nausea, vomiting, diarrhea, and proctitis, were comparable between the two groups, with no statistically significant differences observed.

Genitourinary and skin toxicity: Grade 2 skin reactions were significantly higher in the CCRT arm compared to the AFRT arm (4.5% versus 18.75%; p=0.03). Vaginal mucositis did not differ significantly between groups.

Renal toxicity: Acute kidney injury (AKI) was observed only in the CCRT arm (11/48, 22.9%), with statistical significance (p=0.0007). By grade, this comprised grade 1 AKI in 7 patients (14.6%; p=0.008), grade 2 AKI in 3 patients (6.25%; p=0.09, not statistically significant), and grade 3 AKI in 1 patient (2.1%; p=0.33, not statistically significant) occurring after a single cycle of cisplatin, following which chemotherapy was discontinued in that patient (Table 5).

**Table 5:** Non-hematological acute toxicities AKI: acute kidney injury, AFRT: accelerated fractionation radiotherapy, CCRT: concurrent chemoradiotherapy

Toxicity	AFRT (n=44)	CCRT (n=48)	p-value
Grade ≥2 diarrhea	17 (38.6%)	24 (50%)	0.45
Grade ≥2 skin reactions	2 (4.5%)	9 (18.75%)	0.03
AKI	0 (0%)	11 (22.9%)	0.0007
AKI - grade 1	0 (0%)	7 (14.6%)	0.008
AKI - grade 2	0 (0%)	3 (6.25%)	0.09
AKI - grade 3	0 (0%)	1 (2.1%)	0.33

Late toxicities

Late toxicities were assessed in 24 patients in the AFRT arm and 22 patients in the CCRT arm due to limited follow-up. No statistically significant differences in late toxicity were observed between the two groups.

Given this small subsample, the study has limited power to detect clinically meaningful differences in late toxicity, and these non-significant comparisons should not be interpreted as evidence of equivalence between arms (risk of type II error); 95% confidence intervals are reported in Table 6 to illustrate the width of this uncertainty.

**Table 6:** Late toxicity profile with 95% confidence intervals AFRT: accelerated fractionation radiotherapy, CCRT: concurrent chemoradiotherapy, CI: confidence interval (Wilson score method)

Toxicity	Grade	AFRT (n=24)	95% CI (AFRT)	CCRT (n=22)	95% CI (CCRT)	p-value
Bladder	1	4 (16.7%)	6.7%-35.9%	7 (31.8%)	16.4%-52.7%	0.202
	2	1 (4.1%)	0.7%-20.2%	2 (9.1%)	2.5%-27.8%	
Bowel	1	4 (16.6%)	6.7%-35.9%	7 (14.6%)	16.4%-52.7%	0.217
	2	2 (8.3%)	2.3%-25.8%	3 (13.6%)	4.7%-33.3%	
Skin and subcutaneous	1	3 (12.5%)	4.3%-31.0%	5 (22.7%)	10.1%-43.4%	0.276
	2	0 (0%)	0%-13.8%	1 (4.5%)	0.8%-21.8%	

Bladder Toxicity

Grade 1 cystitis was observed in 16.7% of AFRT patients and 31.8% of CCRT patients, while grade 2 toxicity occurred in 4.1% and 9.1%, respectively. No grade ≥3 toxicities were noted. The differences were not statistically significant.

Bowel Toxicity

Grade 1 proctitis was observed in 16.6% of AFRT patients and 14.6% of CCRT patients, while grade 2 toxicity occurred in 8.3% and 13.6%, respectively. No grade ≥3 toxicities were reported. The differences were not statistically significant.

Skin and Subcutaneous Tissue Toxicity

Grade 1 skin toxicity was observed in 12.5% of AFRT patients and 22.7% of CCRT patients. Grade 2 toxicity was observed in 4.5% of CCRT patients only. No higher-grade toxicities were seen. The differences were not statistically significant.

## Discussion

The present study provides clinically relevant evidence supporting the role of accelerated fractionation radiotherapy (AFRT) as a pragmatic alternative to concurrent chemoradiotherapy (CCRT) in patients with locally advanced cervical carcinoma who are unsuitable for chemotherapy. The key contribution of this study lies in demonstrating that treatment acceleration, without altering total dose, can achieve comparable short-term tumor response while significantly reducing overall treatment time (OTT), a critical determinant of tumor control in cervical cancer. Similar observations have been reported in prospective comparative studies by Sharma et al. [15] and Basu et al. [16], which demonstrated comparable response rates between accelerated radiotherapy and CCRT with a significant reduction in treatment duration in the accelerated arm.

These findings reinforce the radiobiological principle that prolongation of treatment duration adversely impacts tumor control due to accelerated repopulation of clonogenic cells. Multiple clinical studies have consistently demonstrated the detrimental effect of prolonged OTT on local control and survival outcomes in cervical cancer [7,8]. By effectively shortening OTT, the present findings suggest that AFRT may represent a feasible treatment option for carefully selected patients who are unsuitable for concurrent chemotherapy. This is particularly relevant in real-world clinical settings, where a substantial proportion of patients cannot receive cisplatin due to age, renal dysfunction, or comorbid conditions.

Importantly, the study highlights a more favorable acute toxicity profile with AFRT, especially with respect to hematological and renal toxicities, which were significantly higher in the CCRT arm, and this association persisted after age adjustment. This observation is consistent with landmark randomized trials and meta-analyses establishing CCRT as standard of care but also documenting increased hematological toxicity associated with cisplatin-based regimens [4]. Similar trends have been reported in studies by Sharma et al. [15] and Basu et al. [16], which demonstrated higher hematological toxicity in the CCRT arm, although with varying statistical significance. The comparable response rates observed in this study are consistent with earlier reports, including studies by Roy et al. [17], Sharma et al. [15], and Basu et al. [16], which have demonstrated that accelerated schedules can achieve outcomes similar to chemoradiation in selected patient populations. Additionally, prospective evidence has shown that accelerated radiotherapy achieves comparable response rates to chemoradiotherapy with similar toxicity profiles, except for higher renal toxicity in the chemoradiation arm [18], further supporting its role as an alternative in patients unfit for chemotherapy. Gastrointestinal toxicities were comparable between the two approaches, while skin toxicity showed a trend toward higher incidence with CCRT, consistent with existing literature.

In addition, contemporary reviews have emphasized the potential role of altered fractionation in improving tumor control by mitigating treatment prolongation, particularly in settings where chemotherapy is not feasible [19]. However, it is important to interpret these findings cautiously, as the current study evaluated short-term response rather than long-term survival outcomes.

From a clinical perspective, this study adds to the growing body of evidence supporting individualized treatment approaches in cervical cancer, particularly in resource-limited settings or in patients with contraindications to chemotherapy. AFRT offers a feasible strategy to optimize treatment delivery by reducing delays and improving compliance, which are critical factors influencing outcomes in routine practice.

Nevertheless, the study has several important limitations that should be considered when interpreting these findings. First, as treatment allocation was based on clinical fitness rather than randomization, age was a strong confounder (median: 62.5 versus 51 years, with 59% versus 21% of patients over 60). Age-adjusted logistic regression, including Firth's penalized regression for outcomes with zero events in one arm, yielded findings comparable to the unadjusted analyses; however, residual confounding by indication and other unmeasured confounders cannot be excluded because of the non-randomized design and modest sample size. Accordingly, these findings should be interpreted as hypothesis-generating rather than causal. Second, the median follow-up was short and heterogeneous (5 months; range: 2-13 months); therefore, the reported complete response and absence of locoregional recurrence reflect short-term assessment only and do not permit inference regarding progression-free or overall survival. Third, late toxicity was evaluable in only 24/44 (54.5%) AFRT and 22/48 (45.8%) CCRT patients. Consequently, the study was underpowered to detect clinically meaningful differences in late toxicity, and non-significant findings should not be interpreted as evidence of equivalence between treatment arms (limited statistical power to detect differences and potential for a type II error).

## Conclusions

AFRT achieved short-term response rates comparable to CCRT while significantly shortening treatment duration and being associated with lower hematological and renal acute toxicity, suggesting its potential role as a pragmatic alternative for patients unfit for chemotherapy. These associations persisted after age adjustment; however, residual confounding cannot be excluded given the non-randomized design. These findings are limited to short-term response and acute toxicity outcomes and do not permit conclusions regarding progression-free survival, overall survival, or comparative late toxicity profiles. They are particularly relevant in real-world and resource-limited settings, where a substantial proportion of patients may not be suitable candidates for cisplatin-based chemotherapy.

Given the non-randomized design, modest sample size, and short follow-up, these results should be viewed as hypothesis-generating. Larger randomized trials with longer follow-up are needed to confirm the long-term efficacy of AFRT and define its role in individualized cervical cancer treatment.
